# 1,1′:4′,1′′-Terphenyl-2′,5′-dicarb­oxy­lic acid dimethyl sulfoxide-*d*_6_ disolvate

**DOI:** 10.1107/S1600536812012056

**Published:** 2012-03-28

**Authors:** Lucian C. Pop, Marcelo Preite, Juan Manuel Manriquez, Andrés Vega, Ivonne Chavez

**Affiliations:** a Université Paul Sabatier, Laboratoire Hétérochimie Fondamentale et Appliquée, UMR/CNRS 5069, France; b Departamento de Química Orgánica, Facultad de Química, Pontificia Universidad Católica, Santiago, Chile; c Departamento de Química Inorgánica, Facultad de Química, Pontificia Universidad Católica, Santiago, Chile, Casilla 306 Correo 22, Santiago, Chile; d Universidad Andres Bello, Departamento de Ciencias Químicas, Facultad de Ciencias Exactas, Av. República 275 3^er^ Piso, Santiago, Chile; e Centro para el Desarrollo de la Nanociencia y la Nanotecnología, CEDENNA, Chile

## Abstract

The asymmetric unit of the title solvate, C_20_H_14_O_4_·2C_2_D_6_OS, contains half of the substituted terephthalic acid mol­ecule and one solvent mol­ecule. The centroid of the central benzene ring in the acid mol­ecule is coincident with a crystallographic inversion center. Neither the carboxyl nor the phenyl substituents are coplanar with the central aromatic ring, showing dihedral angles of 53.18 (11) and 47.83 (11)°, respectively. The dimethyl sulfoxide solvent mol­ecules are hydrogen bonded to the carb­oxy­lic acid groups.

## Related literature

For the synthesis of the title compound, see: Deuschel (1951 ▶); Ebel & Deuschel (1956 ▶). For similar mol­ecules, see: Tanaka *et al.* (2009 ▶).
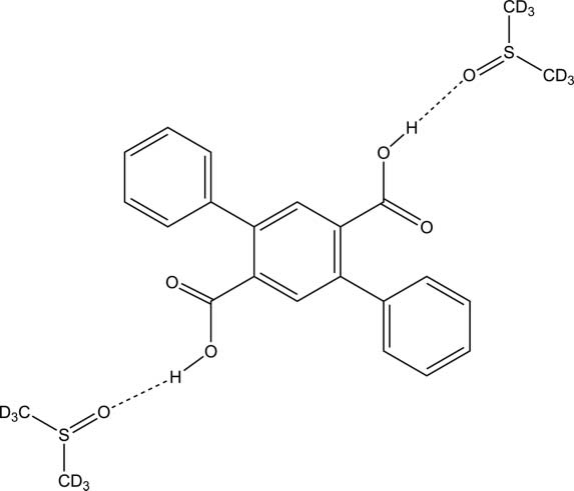


## Experimental

### 

#### Crystal data

C_20_H_14_O_4_·2C_2_D_6_OS
*M* *_r_* = 486.61
Triclinic, 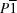

*a* = 6.5184 (9) Å
*b* = 8.8273 (12) Å
*c* = 10.6017 (14) Å
α = 97.076 (2)°
β = 97.074 (2)°
γ = 93.127 (2)°
*V* = 599.26 (14) Å^3^
*Z* = 1
Mo *K*α radiation
μ = 0.26 mm^−1^
*T* = 150 K
0.29 × 0.22 × 0.10 mm

#### Data collection

Siemens SMART CCD area-detector diffractometer
Absorption correction: multi-scan (*SADABS*; Bruker, 2001 ▶) *T*_min_ = 0.929, *T*_max_ = 0.975
3730 measured reflections
2101 independent reflections
1707 reflections with *I* > 2σ(*I*)
*R*_int_ = 0.012

#### Refinement

*R*[*F* ^2^ > 2σ(*F* ^2^)] = 0.049
*wR*(*F* ^2^) = 0.145
*S* = 1.04
2101 reflections
148 parameters
H-atom parameters constrained
Δρ_max_ = 0.53 e Å^−3^
Δρ_min_ = −0.17 e Å^−3^

### 

Data collection: *SMART-NT* (Bruker, 2001 ▶); cell refinement: *SAINT-NT* (Bruker, 1999 ▶); data reduction: *SAINT-NT*; program(s) used to solve structure: *SHELXTL-NT* (Sheldrick, 2008) ▶; program(s) used to refine structure: *SHELXTL-NT* ▶; molecular graphics: *SHELXTL-NT* ▶; software used to prepare material for publication: *SHELXTL-NT* ▶.

## Supplementary Material

Crystal structure: contains datablock(s) I, global. DOI: 10.1107/S1600536812012056/fy2045sup1.cif

Structure factors: contains datablock(s) I. DOI: 10.1107/S1600536812012056/fy2045Isup2.hkl

Supplementary material file. DOI: 10.1107/S1600536812012056/fy2045Isup3.cml

Additional supplementary materials:  crystallographic information; 3D view; checkCIF report

## Figures and Tables

**Table 1:** Hydrogen-bond geometry (Å, °)

*D*—H⋯*A*	*D*—H	H⋯*A*	*D*⋯*A*	*D*—H⋯*A*
O1—H10⋯O3^i^	0.926	1.659	2.581 (2)	174

Symmetry code: (i) 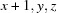.

**Fig. 1.**
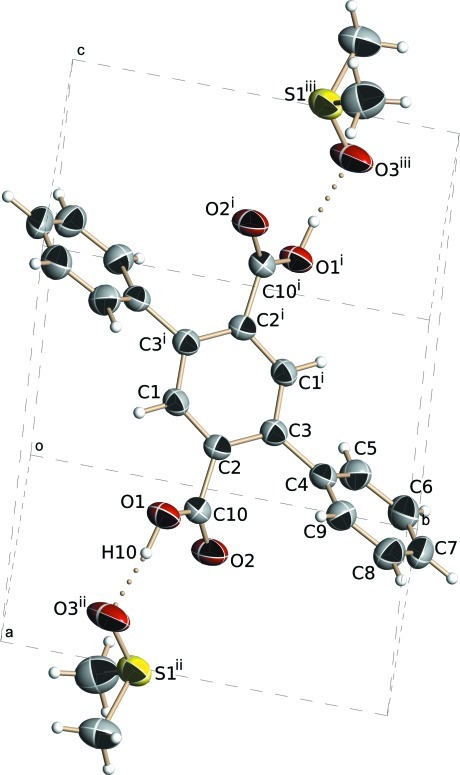
Molecular structure diagram for I, showing the numbering scheme. Displacement ellipsoids are drawn at the 25% probability level and H atoms are shown as spheres of arbitrary radii. Symmetry codes (i): –x+1, –y+1, –z+1; (ii) x + 1, y, z; (iii): –x, –y+1, –z+1.

## supplementary crystallographic information

### Comment

2,5-Diphenyl-1,4-benzenedicarboxylic acid (2,5-Diphenylterephthalic acid) has been described previously as a precursor towards *trans*-fluorenacenedione, prepared by its dehydration with sulfuric acid (Ebel & Deuschel, 1956).

The compound has a central terephtalic acid core, substituted at positions 2 and 5 with phenyl groups. The molecule has been previoulsy described by Tanaka *et al.* (Tanaka *et al.*, 2009) in the form of an ethanol solvate. Tanaka *et al.* also described a related compound where the phenyl groups were replaced by *p*-fluorophenyl groups, which crystallized as a dimethylformamide solvate.

The carboxylic acid and the phenyl groups are highly planar. They define dihedral angles of 53.18 (11)° and 47.83 (11)°, respectively, with the central aromatic ring. The corresponding values are 28.2 (2)° and 57.2 (1)°, and 115.3 (1)° and 46.3 (1)°, for both dihedral angles in the ethanol solvate, and in the *p*-fluoro compound, respectively.

The molecule has an inversion center (crystallographic) coincident with the centroid of the central ring, so the point group symmetry of the isolated molecule is C_i_. The same happens for the ethanol solvate and the *p*-fluoro compounds respectively.

The packing shows a deuterated dimethyl sulfoxide solvent molecule hydrogen bonded with each carboxylic acid group, with O1···O3^ii^ distance of 2.581 (2) Å (ii: *x* + 1, *y*, *z*). There are two molecules of solvent for a single diacid molecule, each one defining one of the aforementioned hydrogen bond with each carboxylic acid group. A closely related pattern occurs for the ethanol solvate of the title molecule and the dimethylformamide solvate of the *p*-fluoro derivative (Tanaka *et al.*, 2009): two molecules of solvent, ethanol or dimethylformamide, are bonded by hydrogen bonds to both carboxylic acid groups.

### Experimental

The compound was prepared by a method described in the literature (Deuschel, 1951; Ebel & Deuschel, 1956), slighty modified by using d_6_-DMSO for crystallization instead of C_6_H_5_CN, giving the DMSO-clathrate. The title compound was prepared in a 93% yield, mp. = 280°C (dec).

### Refinement

The hydrogen atoms positions were calculated after each cycle of refinement with *SHELXL* (Bruker,1999) using a riding model, with C—H distances in the range of 0.93 to 0.96 Å. *U*_iso_(H) values were set equal to 1.5*U*_eq_ of the parent carbon atom for methyl groups, and 1.2*U*_eq_ for the others. The carboxylic acid hydrogen atom was located in the difference Fourier map, and its coordinates were subsequentely kept fixed (by adding 10 to the coordinates in SHELXL), while *U*_iso_(H) was left free to refine.

### Crystal data

**Table:**

C_20_H_14_O_4_·2C_2_D_6_OS	*Z* = 1
*M**_r_* = 486.61	*F*(000) = 250
Triclinic, *P*1	*D*_x_ = 1.348 Mg m^−^^3^
Hall symbol: -P 1	Mo *K*α radiation, λ = 0.71073 Å
*a* = 6.5184 (9) Å	Cell parameters from 1643 reflections
*b* = 8.8273 (12) Å	θ = 2.3–24.2°
*c* = 10.6017 (14) Å	µ = 0.26 mm^−^^1^
α = 97.076 (2)°	*T* = 150 K
β = 97.074 (2)°	Block, colorless
γ = 93.127 (2)°	0.29 × 0.22 × 0.10 mm
*V* = 599.26 (14) Å^3^	

### Data collection

**Table:**

Siemens SMART CCD area-detector diffractometer	2101 independent reflections
Radiation source: fine-focus sealed tube	1707 reflections with *I* > 2σ(*I*)
Graphite monochromator	*R*_int_ = 0.012
φ and ω scans	θ_max_ = 25.0°, θ_min_ = 2.3°
Absorption correction: multi-scan (*SADABS*; Bruker, 2001)	*h* = −7→7
*T*_min_ = 0.929, *T*_max_ = 0.975	*k* = −10→10
3730 measured reflections	*l* = −12→12

### Refinement

**Table:**

Refinement on *F*^2^	Primary atom site location: structure-invariant direct methods
Least-squares matrix: full	Secondary atom site location: difference Fourier map
*R*[*F*^2^ > 2σ(*F*^2^)] = 0.049	Hydrogen site location: inferred from neighbouring sites
*wR*(*F*^2^) = 0.145	H-atom parameters constrained
*S* = 1.04	*w* = 1/[σ^2^(*F*_o_^2^) + (0.0891*P*)^2^ + 0.1414*P*] where *P* = (*F*_o_^2^ + 2*F*_c_^2^)/3
2101 reflections	(Δ/σ)_max_ < 0.001
148 parameters	Δρ_max_ = 0.53 e Å^−^^3^
0 restraints	Δρ_min_ = −0.17 e Å^−^^3^

### Special details

**Table:**

Geometry. All e.s.d.'s (except the e.s.d. in the dihedral angle between two l.s. planes) are estimated using the full covariance matrix. The cell e.s.d.'s are taken into account individually in the estimation of e.s.d.'s in distances, angles and torsion angles; correlations between e.s.d.'s in cell parameters are only used when they are defined by crystal symmetry. An approximate (isotropic) treatment of cell e.s.d.'s is used for estimating e.s.d.'s involving l.s. planes.
Refinement. Refinement of *F*^2^ against ALL reflections. The weighted *R*-factor *wR* and goodness of fit *S* are based on *F*^2^, conventional *R*-factors *R* are based on *F*, with *F* set to zero for negative *F*^2^. The threshold expression of *F*^2^ > σ(*F*^2^) is used only for calculating *R*-factors(gt) *etc*. and is not relevant to the choice of reflections for refinement. *R*-factors based on *F*^2^ are statistically about twice as large as those based on *F*, and *R*- factors based on ALL data will be even larger.

### Fractional atomic coordinates and isotropic or equivalent isotropic displacement parameters (Å^2^)

**Table:**

	*x*	*y*	*z*	*U*_iso_*/*U*_eq_	
C1	0.6023 (3)	0.3696 (2)	0.48626 (19)	0.0397 (5)	
H1	0.6737	0.2814	0.4770	0.048*	
C2	0.6546 (3)	0.4883 (2)	0.41945 (19)	0.0371 (5)	
C10	0.8077 (3)	0.4593 (2)	0.3263 (2)	0.0408 (5)	
O1	0.9582 (2)	0.37837 (18)	0.36916 (14)	0.0503 (4)	
O2	0.7903 (3)	0.5003 (2)	0.22114 (16)	0.0603 (5)	
H10	1.0348	0.3461	0.3042	0.073 (8)*	
C3	0.5515 (3)	0.6244 (2)	0.43352 (19)	0.0385 (5)	
C4	0.6018 (3)	0.7627 (2)	0.3727 (2)	0.0413 (5)	
C5	0.4423 (4)	0.8397 (3)	0.3156 (2)	0.0532 (6)	
H5	0.3059	0.8007	0.3118	0.064*	
C6	0.4828 (5)	0.9733 (3)	0.2644 (3)	0.0637 (7)	
H6	0.3746	1.0236	0.2265	0.076*	
C7	0.6846 (5)	1.0312 (3)	0.2699 (3)	0.0640 (7)	
H7	0.7132	1.1199	0.2343	0.077*	
C8	0.8446 (4)	0.9578 (3)	0.3283 (2)	0.0607 (7)	
H8	0.9807	0.9980	0.3329	0.073*	
C9	0.8033 (4)	0.8244 (3)	0.3801 (2)	0.0500 (6)	
H9	0.9118	0.7760	0.4200	0.060*	
S1	0.18071 (11)	0.35077 (8)	0.07652 (6)	0.0630 (3)	
O3	0.1731 (3)	0.2709 (3)	0.19405 (18)	0.0834 (7)	
C11	0.4021 (4)	0.2871 (4)	0.0103 (3)	0.0744 (8)	
D11A	0.5249	0.3310	0.0647	0.112*	
D11B	0.4020	0.3183	−0.0733	0.112*	
D11C	0.3993	0.1776	0.0035	0.112*	
C12	−0.0093 (5)	0.2526 (4)	−0.0412 (3)	0.0850 (10)	
D12A	0.0088	0.1449	−0.0479	0.127*	
D12B	0.0036	0.2878	−0.1222	0.127*	
D12C	−0.1442	0.2715	−0.0180	0.127*	

### Atomic displacement parameters (Å^2^)

**Table:**

	*U*^11^	*U*^22^	*U*^33^	*U*^12^	*U*^13^	*U*^23^
C1	0.0418 (11)	0.0386 (11)	0.0396 (11)	0.0091 (8)	0.0070 (9)	0.0044 (9)
C2	0.0362 (10)	0.0422 (11)	0.0333 (10)	0.0052 (8)	0.0054 (8)	0.0045 (8)
C10	0.0433 (11)	0.0400 (11)	0.0397 (12)	0.0049 (9)	0.0094 (9)	0.0030 (9)
O1	0.0478 (9)	0.0628 (10)	0.0453 (9)	0.0194 (7)	0.0156 (7)	0.0105 (7)
O2	0.0705 (11)	0.0738 (12)	0.0461 (10)	0.0263 (9)	0.0230 (8)	0.0208 (8)
C3	0.0386 (11)	0.0411 (11)	0.0359 (11)	0.0049 (8)	0.0046 (8)	0.0050 (8)
C4	0.0470 (12)	0.0416 (12)	0.0377 (11)	0.0087 (9)	0.0115 (9)	0.0065 (9)
C5	0.0520 (13)	0.0555 (15)	0.0569 (14)	0.0134 (11)	0.0118 (11)	0.0177 (11)
C6	0.0789 (18)	0.0539 (15)	0.0655 (17)	0.0245 (13)	0.0157 (14)	0.0211 (12)
C7	0.098 (2)	0.0383 (13)	0.0591 (16)	0.0018 (13)	0.0223 (14)	0.0104 (11)
C8	0.0687 (16)	0.0521 (15)	0.0605 (16)	−0.0109 (12)	0.0133 (13)	0.0055 (12)
C9	0.0497 (13)	0.0495 (13)	0.0519 (14)	0.0036 (10)	0.0091 (10)	0.0090 (10)
S1	0.0719 (5)	0.0675 (5)	0.0573 (5)	0.0266 (3)	0.0223 (3)	0.0148 (3)
O3	0.1020 (16)	0.1079 (16)	0.0601 (12)	0.0620 (13)	0.0431 (11)	0.0331 (11)
C11	0.0644 (17)	0.102 (2)	0.0625 (17)	0.0194 (16)	0.0214 (14)	0.0155 (16)
C12	0.0634 (18)	0.104 (2)	0.091 (2)	0.0067 (17)	0.0121 (16)	0.0226 (19)

### Geometric parameters (Å, º)

**Table:**

C1—C2	1.385 (3)	C6—H6	0.9300
C1—C3^i^	1.391 (3)	C7—C8	1.380 (4)
C1—H1	0.9300	C7—H7	0.9300
C2—C3	1.410 (3)	C8—C9	1.386 (3)
C2—C10	1.498 (3)	C8—H8	0.9300
C10—O2	1.209 (3)	C9—H9	0.9300
C10—O1	1.314 (3)	S1—O3	1.5101 (19)
O1—H10	0.926	S1—C12	1.754 (3)
C3—C1^i^	1.391 (3)	S1—C11	1.769 (3)
C3—C4	1.489 (3)	C11—D11A	0.9600
C4—C9	1.384 (3)	C11—D11B	0.9600
C4—C5	1.390 (3)	C11—D11C	0.9600
C5—C6	1.384 (3)	C12—D12A	0.9600
C5—H5	0.9300	C12—D12B	0.9600
C6—C7	1.377 (4)	C12—D12C	0.9600
			
C2—C1—C3^i^	123.41 (19)	C6—C7—H7	120.0
C2—C1—H1	118.3	C8—C7—H7	120.0
C3^i^—C1—H1	118.3	C7—C8—C9	120.2 (2)
C1—C2—C3	119.46 (18)	C7—C8—H8	119.9
C1—C2—C10	117.34 (18)	C9—C8—H8	119.9
C3—C2—C10	122.99 (19)	C4—C9—C8	120.4 (2)
O2—C10—O1	123.55 (19)	C4—C9—H9	119.8
O2—C10—C2	123.75 (19)	C8—C9—H9	119.8
O1—C10—C2	112.63 (18)	O3—S1—C12	105.90 (15)
C10—O1—H10	110.50	O3—S1—C11	105.11 (13)
C1^i^—C3—C2	117.12 (19)	C12—S1—C11	98.15 (16)
C1^i^—C3—C4	118.39 (18)	S1—C11—D11A	109.5
C2—C3—C4	124.45 (18)	S1—C11—D11B	109.5
C9—C4—C5	118.6 (2)	D11A—C11—D11B	109.5
C9—C4—C3	121.63 (19)	S1—C11—D11C	109.5
C5—C4—C3	119.6 (2)	D11A—C11—D11C	109.5
C6—C5—C4	121.2 (2)	D11B—C11—D11C	109.5
C6—C5—H5	119.4	S1—C12—D12A	109.5
C4—C5—H5	119.4	S1—C12—D12B	109.5
C7—C6—C5	119.5 (2)	D12A—C12—D12B	109.5
C7—C6—H6	120.2	S1—C12—D12C	109.5
C5—C6—H6	120.2	D12A—C12—D12C	109.5
C6—C7—C8	120.1 (2)	D12B—C12—D12C	109.5
			
C3^i^—C1—C2—C3	1.1 (3)	C2—C3—C4—C9	−48.8 (3)
C3^i^—C1—C2—C10	−173.80 (19)	C1^i^—C3—C4—C5	−46.1 (3)
C1—C2—C10—O2	137.8 (2)	C2—C3—C4—C5	136.4 (2)
C3—C2—C10—O2	−37.0 (3)	C9—C4—C5—C6	1.4 (4)
C1—C2—C10—O1	−39.3 (3)	C3—C4—C5—C6	176.4 (2)
C3—C2—C10—O1	145.9 (2)	C4—C5—C6—C7	0.0 (4)
C1—C2—C3—C1^i^	−1.1 (3)	C5—C6—C7—C8	−1.2 (4)
C10—C2—C3—C1^i^	173.57 (18)	C6—C7—C8—C9	0.9 (4)
C1—C2—C3—C4	176.46 (19)	C5—C4—C9—C8	−1.8 (3)
C10—C2—C3—C4	−8.9 (3)	C3—C4—C9—C8	−176.6 (2)
C1^i^—C3—C4—C9	128.7 (2)	C7—C8—C9—C4	0.6 (4)

Symmetry code: (i) −*x*+1, −*y*+1, −*z*+1.

### Hydrogen-bond geometry (Å, º)

**Table:**

*D*—H···*A*	*D*—H	H···*A*	*D*···*A*	*D*—H···*A*
O1—H10···O3^ii^	0.926	1.659	2.581 (2)	174

Symmetry code: (ii) *x*+1, *y*, *z*.
